# Freestanding PZT Films for Integrated Wearable Sensing and Intelligent Acoustic Recognition

**DOI:** 10.1002/advs.78091

**Published:** 2026-10-14

**Authors:** Hailong Pan, Zhichao Xu, Rongxiao Liu, Jianmei Pei, Changhong Yang, Shifeng Huang, Michael Wübbenhorst

**Affiliations:** ^1^ Shandong Provincial Key Laboratory of Green and Intelligent Building Materials University of Jinan Jinan People's Republic of China; ^2^ Laboratory for Soft Matter and Biophysics Department of Physics and Astronomy KU Leuven Leuven Belgium

**Keywords:** freestanding epitaxial PZT thin films, intelligent acoustic recognition, substrate unclamping, water‐assisted delamination, wearable sensing

## Abstract

Flexible, self‐powered sensors are pivotal for next‐generation human‐machine interfaces. However, substrate‐induced clamping fundamentally restricts their electromechanical performance by suppressing longitudinal deformation, thereby reducing signal sensitivity. Here, we report a high‐performance freestanding PbZr_0.52_Ti_0.48_O_3_ (PZT) thin film based on a layered Mica/Pt/LaNiO_3_ heterostructure. The complete release of substrate constraints unlocks markedly enhanced properties, including a 1.4‐fold increase in remanent polarization (*P*_r_ = 54 vs. 40 µC cm^−^^2^), a 2.2‐fold enhancement in the effective converse piezoelectric coefficient (*d*_33_ = 390 pm V^−1^ vs. 174 pm V^−1^), and an approximate 1.5‐fold improvement in dielectric permittivity. Consequently, the device exhibits rapid electromechanical transduction with an ultrashort pulse duration of 500 µs, alongside high output voltage and power. Integrated onto the human body, the sensor delivers sensitive, repeatable responses to various biomechanical motions. Beyond tactile sensing, the device enables high‐fidelity acoustic detection and intelligent signal analysis. By implementing harmonic evaluation and principal component analysis, the system achieves reliable discrimination among musical instruments with distinctive spectral fingerprints. Collectively, this work establishes the freestanding PZT film as a multifunctional platform integrating energy harvesting and intelligent acoustic recognition, offering new pathways for self‐powered biointegrated electronics.

## Graphical abstract

A freestanding epitaxial PZT/LaNiO_3_/Pt heterostructure is achieved via water‐assisted delamination from mica, effectively eliminating substrate‐induced clamping and releasing intrinsic ferroelectricity. The released film exhibits substantially enhanced remanent polarization and piezoelectric response, delivering excellent mechanical flexibility and long‐term operational stability. Such freestanding PZT films enable highly integrated wearable sensing and intelligent acoustic recognition for accurate music signal classification.

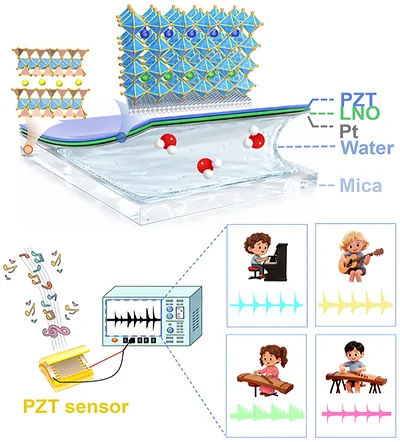

## 1 Introduction

Flexible piezoelectric materials have attracted significant attention for next‐generation intelligent systems, including self‐powered sensors, wearable electronics, and human‐machine interfaces owing to their reversible electromechanical conversion capability [1, 2, 3, 4]. Compared to organic counterparts, inorganic piezoelectric thin films offer superior piezoelectric responses, thermal stability, and operational durability, enabling efficient energy harvesting and reliable device operation [5, 6, 7]. Among various candidates, oxide‐based systems stand out for their large spontaneous polarization, strong electromechanical coupling, and superior stability. In particular, when integrated into flexible and wearable platforms, they enable self‐powered detection of acoustic stimuli generated by human activities and environmental sounds [8, 9, 10, 11]. However, most reported systems remain limited to signal acquisition and basic waveform monitoring, with insufficient capability for frequency‐domain feature extraction and reliable recognition of complex acoustic sources. This limits their ability to discriminate sound sources with distinct spectral signatures, such as musical instruments.

As a prototypical ferroelectric perovskite oxide, lead zirconate titanate provides enhanced piezoelectricity near the morphotropic phase boundary (MPB), where competing ferroelectric phases coexist, facilitating polarization rotation [12, 13]. Among them, PbZr_0.52_Ti_0.48_O_3_ (PZT), a representative MPB composition, exhibits large spontaneous polarization, high Curie temperature, and outstanding electromechanical performance [14]. In conventional configurations, PZT thin films are typically integrated on rigid substrates, where strong mechanical clamping severely restricts lattice deformation and intrinsically suppresses electromechanical coupling [15, 16, 17, 18, 19, 20, 21, 22, 23]. To mitigate this limitation, flexible substrates have been introduced to provide enhanced deformability, resulting in an increased piezoelectric response [24, 25, 26, 27]. However, such improvements remain inherently limited, as substrate‐induced clamping cannot be fundamentally eliminated. To address this challenge, flexible free‐standing piezoelectric thin films have emerged as a promising alternative, in which the complete removal of the substrate enables the full release of mechanical constraints. Considerable efforts have been devoted to achieving such structures through either substrate‐assisted fabrication or direct substrate‐free manufacturing [28, 29, 30, 31, 32, 33, 34, 35]. Direct substrate‐free approaches simplify fabrication by eliminating the need for growth on a substrate and subsequent release processes. By comparison, substrate‐assisted growth offers greater control over crystallization, epitaxial orientation, and heterostructure engineering, which is particularly advantageous for high‐performance PZT thin films. Compared with sacrificial layer‐assisted release, which requires additional sacrificial‐layer fabrication and removal steps, water‐assisted delamination provides a simpler and more direct route for releasing the film by exploiting a weakly bonded interface. This delamination strategy has recently been explored for freestanding piezoceramic films, but the reported demonstrations focus on lead‐free compositions or rely on different electrode configurations without the combined Pt/LaNiO_3_ (LNO) buffer architecture [28, 29, 36]. These studies establish the feasibility of water‐assisted delamination, while its application to epitaxial PZT heterostructures with a Pt/LNO architecture remains unexplored. Therefore, developing a facile and effective strategy to simultaneously achieve mechanical compliance and high piezoelectric performance in PZT thin films remains highly desirable.

In this regard, layered mica substrates provide a unique opportunity. Owing to its atomically smooth surface, weak van der Waals interlayer interactions, and thermal stability, mica provides a favourable platform for oxide thin‐film growth with minimized lattice constraints [37, 38]. By integrating functional buffer layers, both electrical performance and crystalline quality can be optimized while preserving releasability. Specifically, the Pt interlayer not only functions as a bottom electrode but also suppresses interdiffusion to stabilize the interface during high‐temperature processing, while its mechanical compliance facilitates subsequent film release. Meanwhile, LNO serves as a conductive, lattice‐matched perovskite template that enables the oriented growth of high‐quality PZT films [39]. These features make such layered‐substrate systems with functional buffer layers particularly attractive for constructing mechanically releasable piezoelectric films with both high crystalline quality and improved structural integrity. Distinct from previous water‐assisted delamination approaches that rely on single metal electrodes or sacrificial layers, our Pt/LNO bilayer architecture simultaneously ensures electrical conductivity, lattice matching, and a releasable Pt/mica interface.

Here, we report a facile water‐assisted delamination strategy to fabricate PZT thin films based on a mica/Pt/LNO/PZT heterostructure. Enabled by the weak van der Waals interaction of mica, the film stack can be readily released, effectively alleviating substrate‐induced mechanical constraints. This approach combines efficient strain release with mechanical robustness. As a result, the obtained PZT film exhibits an enhanced electromechanical response, enabling high‐fidelity detection under pressure‐driven wearable operation and further allowing reliable recognition of musical instruments from single‐frequency, multi‐frequency, and complex musical inputs, highlighting its potential for flexible sensing and energy harvesting applications.

## 2 Results and Discussion

### 2.1 Microstructure and Ferroelectric Properties of Freestanding Films

Figure 1a schematically illustrates the layered architecture of the freestanding PZT film, comprising a PZT functional layer, an LNO buffer layer, and a Pt bottom electrode, which are released from the mica substrate. Unless otherwise specified, the Pt/LNO/PZT heterostructures discussed throughout this work contain an 8‐layer PZT film. The Pt layer functions as both a conductive electrode and a diffusion barrier, while the LNO layer, owing to its good lattice matching with PZT, promotes the epitaxial growth of the PZT film. As a result of the weak interfacial van der Waals interactions between Pt and mica, the heterostructure can be exfoliated through water‐assisted interfacial separation, yielding a freestanding PZT film. This configuration effectively releases the substrate‐induced mechanical constraint. Consequently, beyond the enhanced converse piezoelectric coefficient (*d*_33_) derived from piezoresponse force microscopy (PFM), the freestanding film also exhibits an increased dielectric permittivity (*ε*_r_), an enhanced remanent polarization (*P*_r_), a higher saturation polarization (*P*_s_), and a reduced coercive field (*E*_c_) (Figure 1b). The strong electromechanical coupling in the freestanding film enables efficient electrical output under mechanical stimuli. To elucidate the underlying mechanism, the working principle is illustrated in Figure 1c. Before poling, the dipoles are randomly oriented but progressively align under an applied DC electric field. After poling, the induced polarization is compensated by surface charges on the interdigitated electrodes (IDEs). Mechanical bending induces tensile strain that enhances polarization, driving charge redistribution to screen the piezoelectric potential, while release reverses this process and generates alternating electrical signals. These dynamic electrical outputs can be further analysed in the frequency domain via Fourier transform to extract characteristic spectral features. Quantitative analyses, including spectral centroid extraction and principal component analysis (PCA), are employed to enable reliable discrimination among different instruments and musical notes, as presented in Figure 1d.

**FIGURE 1:**
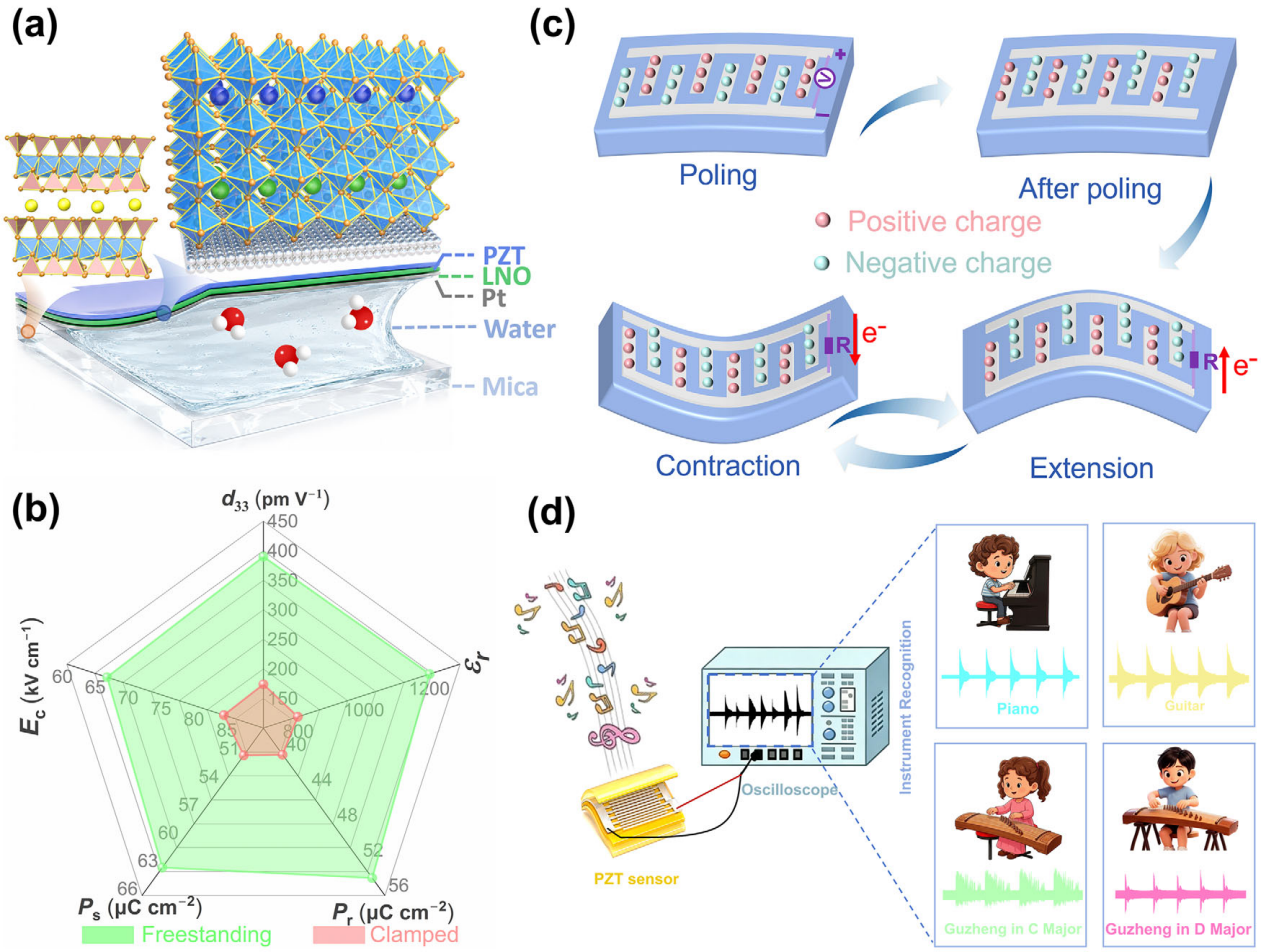
(a) Schematic illustration of the layered structure of the freestanding PZT film. (b) Comparison of the piezoelectric, ferroelectric, and dielectric properties of freestanding and clamped Pt/LNO/PZT heterostructures with an 8‐layer PZT film. (c) Working principle of the flexible sensor. (d) Demonstration of self‐powered acoustic sensing for musical instrument recognition.

To verify the formation of the layered structure, X‐ray diffraction (XRD) measurements were performed. Figure 2a shows XRD patterns of the Pt/LNO/PZT heterostructure. The well‐defined diffraction peaks from each layer confirm the construction of a layered architecture. Meanwhile, the absence of impurity peaks indicates high phase purity, and the disappearance of mica‐related diffraction signals confirms complete exfoliation of the film from the mica substrate. To gain insight into the microstructure, the top‐view scanning electron microscopy (SEM) image of the film was obtained, as illustrated in Figure 2b. A uniform surface morphology with well‐defined grains is observed, exhibiting a narrow grain size distribution centred at ∼30 nm, as confirmed by statistical analysis shown in the inset of Figure 2b. Cross‐sectional SEM (Figure 2c) provides direct evidence of the layered architecture, where the PZT, LNO, and Pt layers are distinguishable, consistent with the designed heterostructure. Energy‐dispersive X‐ray spectroscopy (EDS) elemental mapping further corroborates the layered architecture by revealing the distinct spatial distribution of the constituent elements (Figure S1). The well‐defined layered structure and uniform grain size distribution provide a favourable structural basis for excellent piezoelectric and ferroelectric properties.

**FIGURE 2:**
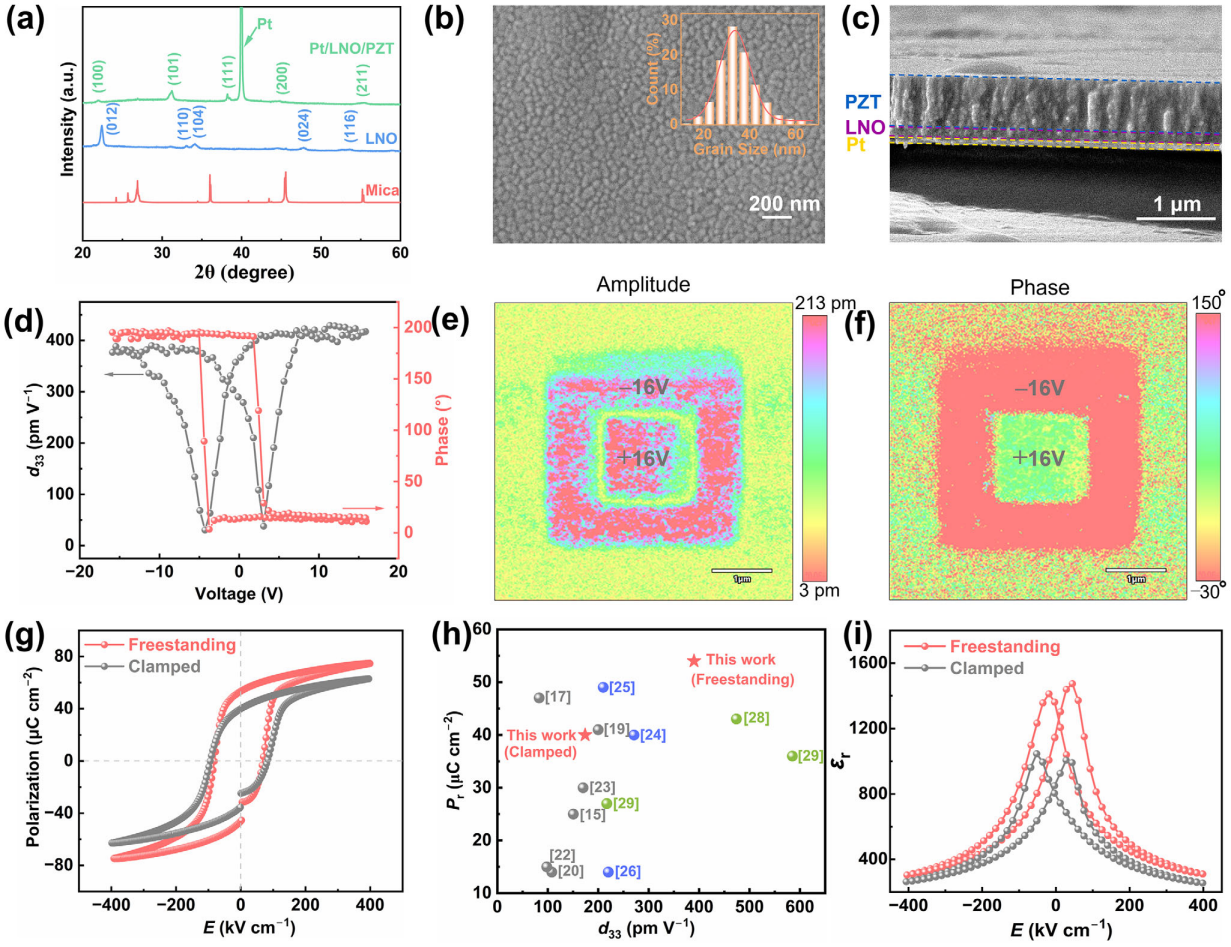
(a) XRD patterns of the Pt/LNO/PZT heterostructure. (b) Top‐view SEM image of the PZT film. (c) Cross‐sectional SEM image of the heterostructure. (d) *d*_33_ amplitude and phase as a function of DC bias for the freestanding film. (e,f) Out‐of‐plane PFM amplitude and phase images of the freestanding PZT film after applying local +16 V/–16 V DC voltage. (g) *P*–*E* loops of freestanding and clamped films. (h) Comparison of *P*_r_ and *d*_33_ for PZT thin films reported in the literature. Note: Grey, blue, and green dots denote the *d*_33_ values of films on rigid substrates, films on flexible substrates, and freestanding films, respectively. (i) *ε*_r_ as a function of electric field for clamped and freestanding films.

To gain insight into the local functional properties, the piezoelectric response and ferroelectric domain switching behaviour were systematically studied by PFM. Figure 2d presents the *d*_33_ amplitude and phase loops of the freestanding PZT film. The characteristic butterfly‐shaped *d*_33_ loop and the nearly 180° phase reversal are characteristic signatures of the piezoelectric response and reversible ferroelectric polarization switching, respectively. Remarkably, the freestanding film exhibits a *d*_33_ of 390 pm V^−1^, approximately 2.2 times higher than that of the clamped counterpart, as demonstrated in Figure S2. Notably, the pronounced enhancement of *d*_33_ for the freestanding film arises from two synergistic mechanisms. First, the release of substrate clamping induces lattice evolution, including the relaxation of misfit strain, which intrinsically improves the intrinsic piezoelectric response. Second, more fundamentally, the removal of the rigid substrate profoundly alters the domain wall dynamics. In clamped films, the mechanical constraint from the substrate pins domain walls, raising the energy barrier for lateral wall motion and suppressing the extrinsic contribution to the piezoelectric effect. In contrast, the freestanding configuration eliminates this pinning interface, allowing domain walls to move more freely under an applied electric field. As a result, the energy barrier for lateral domain wall motion is substantially reduced, and the extrinsic contribution—which is known to dominate the macroscopic piezoelectric response in polycrystalline ferroelectrics—is fully released. This enhanced domain wall mobility not only facilitates polarization switching (as evidenced by the reduced coercive field *E*_c_ in Figure 2g) but also directly contributes to the markedly increased *d*_33_ value (390 pm V^−1^, 2.2‐fold higher than that of the substrate‐clamped film). Such a mechanistic understanding, which goes beyond the conventional lattice‐strain picture, highlights that substrate‐free architectures unlock the full electromechanical potential of ferroelectric thin films by harnessing both intrinsic and extrinsic contributions [40, 41, 42, 43]. As shown in Figure 2e,f, well‐defined box‐in‐box patterns can be successfully written in both the amplitude and phase images by applying opposite DC bias voltages. The pronounced amplitude contrast and nearly 180° phase contrast between oppositely polarized domains confirm reversible local polarization switching, demonstrating a clear ferroelectric response at the microscale.

To complement the local PFM characterization, the macroscopic ferroelectric behaviour was evaluated using polarization–electric field (*P*–*E*) hysteresis loops. Compared with the clamped counterpart, the freestanding film exhibits significantly enhanced *P*_r_ (54 vs. 40 µC cm^−2^) and *P*_s_ (63 vs. 51 µC cm^−2^), along with a reduced *E*_c_ (66 vs. 84 kV cm^−^^1^), as exhibited in Figure 2g. To further elucidate the ferroelectric behaviour, Figure S3 displays a comprehensive evaluation of the freestanding film under varied electrical and cycling conditions. As shown in Figure S3a, the *P*–*E* loops progressively expand with increasing applied voltage, indicating enhanced polarization switching under stronger driving fields. Figure S3b evaluates the frequency‐dependent ferroelectric response, where the *P*–*E* loops remain well‐saturated across the measured frequency range, confirming stable switching. Furthermore, Figure S3c demonstrates excellent polarization cycling stability over 10^9^ switching cycles, with only slight degradation, indicating robust fatigue resistance. Consistently, Figure S3d compares the *P*–*E* loops before and after 10^9^ cycles, where the ferroelectric response is largely retained, further confirming the excellent cycling reliability of the films. To further compare the ferroelectric responses across different PZT thicknesses, Pt/LNO/PZT heterostructures with 6‐, 8‐, and 10‐layer PZT films are investigated under both clamped and freestanding conditions (Figure S4a–c). In both configurations, increasing the PZT thickness generally results in higher *P*_r_ and *P*_s_ and a lower *E*_c_. Despite these thickness‐dependent variations, the freestanding heterostructures consistently exhibit enhanced ferroelectric responses compared with their clamped counterparts at all three investigated thicknesses.

*d*_33_ and *P*_r_ are two key parameters for evaluating the electromechanical and ferroelectric performance of PZT‐based thin films, respectively. To provide a comprehensive comparison, representative literature values of *d*_33_ and *P*_r_ are summarized in Tables S1 and plotted in Figure 2h. The grey, blue, and green dots denote the *d*_33_ values of films on rigid substrates, films on flexible substrates, and freestanding films, respectively. Interestingly, in clamped ferroelectric thin films, the positive correlation between *d*_33_ and *P*_r_ originates from their shared dependence on switchable polarization magnitude and domain reorientation capability under substrate‐constrained strain conditions. In addition, releasing substrate clamping relaxes epitaxial strain constraints, enabling a more readily reconfigurable ferroelectric domain state with enhanced polarization alignment and domain‐wall mobility, thereby simultaneously enhancing both *d*_33_ and *P*_r_. Notably, our sample simultaneously achieves the highest *P*_r_ and a moderate *d*_33_, demonstrating an optimal balance between polarization stability and electromechanical response, thereby delivering superior overall ferroelectric functionality. To investigate the effect of substrate release on the dielectric response, the dielectric properties of the films are investigated. Figure 2i shows that *ε*_r_ of the freestanding film (1207) is approximately 1.5‐fold that of the clamped counterpart (805), reflecting enhanced dielectric susceptibility resulting from the release of substrate‐induced mechanical constraints. To further examine the dielectric response at different PZT thicknesses, Pt/LNO/PZT heterostructures with 6‐, 8‐, and 10‐layer PZT films are evaluated under both clamped and freestanding conditions (Figure S4d–f). Although *ε*_r_ varies with PZT thickness, the freestanding heterostructures consistently exhibit higher *ε*_r_ than their clamped counterparts at all three thicknesses. Together with the enhanced polarization response observed at the corresponding thicknesses (Figure S4a–c), these results demonstrate consistently improved dielectric and ferroelectric properties in the freestanding PZT films, further supporting the beneficial effect of substrate release.

### 2.2 Mechanical Energy Harvesting and Wearable Sensing Performance

With the significantly improved piezoelectric response of the freestanding film, we next integrated it into a flexible sensor configuration to evaluate its electromechanical energy conversion capability. IDEs were deposited on top of the PZT layer, and the entire device was encapsulated with polyimide (PI) tape for mechanical protection and electrical insulation (inset of Figure 3d). To evaluate the mechanical energy‐harvesting capability of the sensor, an impact‐testing setup was employed, in which periodic impact excitation was induced by a freely falling mass, as illustrated in Figure 3a. Under impact loading, the output voltage increases with the dropped mass (2–20 g), indicating a strong correlation between the electrical response and the impact energy (Figure 3b). The sensor exhibits high sensitivity, generating an output voltage of up to 2.5 V even under a small impact load of 2 g, indicating excellent mechanical‐to‐electrical transduction capability. Importantly, under identical impact conditions, the freestanding film generates a substantially higher output voltage than the clamped counterpart, demonstrating enhanced electromechanical transduction resulting from the release of substrate‐induced clamping (Figure 3c). The transient response exhibits a sharp voltage pulse with a response time of approximately 500 µs, indicating rapid electromechanical transduction and excellent dynamic response (Figure 3d). This response time is substantially shorter than the previously reported 27 ms for Sm‐doped PMN–PT films [44].

**FIGURE 3:**
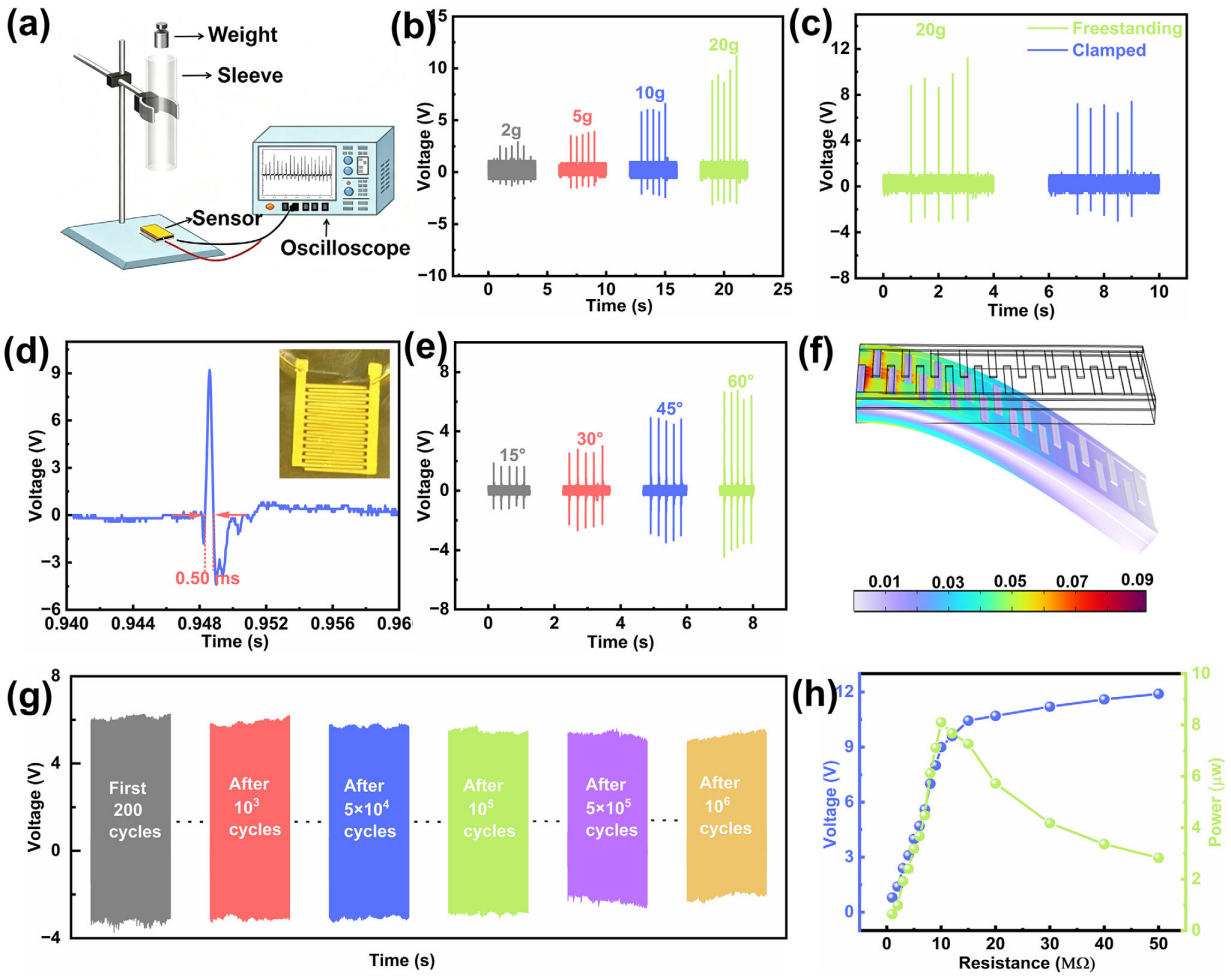
(a) Schematic illustration of the impact measurement setup using a free‐falling mass. (b) Output voltage of the freestanding film under impact from masses of different weights dropped from a height of 20 cm. (c) Comparison of output voltage responses between clamped and freestanding films under impact from a 20 g mass dropped from a height of 20 cm. (d) Magnified transient output voltage response under impact excitation. Inset: Photograph of the encapsulated device. (e) Output voltage of the sensor under bending deformation at different angles (15°, 30°, 45°, and 60°). (f) Finite element simulation of the strain distribution in the sensor under 30° bending. (g) Cyclic durability of the flexible sensor. (h) Output voltage and calculated output power versus external load resistance.

In addition to impact excitation, the sensor generates robust voltage outputs under bending deformation, demonstrating efficient electromechanical transduction under dynamic mechanical stimuli. Furthermore, the output voltage increases monotonically with bending angle (from 15° to 60°), demonstrating a strong deformation‐dependent electromechanical response, as presented in Figure 3e. The maximum output voltage at a bending angle of 60° reaches approximately 7.5 V, which is substantially higher than that of reported PZT‐based sensors (1.5 V) and powder‐based PZT films (2 V) [27, 45]. Figure S5 shows the output voltage of the clamped film under a 60° bending deformation. The markedly suppressed electrical response highlights the critical role of the freestanding configuration in enabling enhanced voltage generation. In addition, finite element analysis (FEA) was performed to visualize and quantify strain distributions under different bending angles. Figure 3f illustrates the strain distribution of the sensor at a bending angle of 30°, revealing that the strain is predominantly concentrated at the fixed end of the cantilever. Moreover, the strain distribution becomes more pronounced with increasing bending angle (Figure S6), consistent with the experimental results. Beyond instantaneous performance, the sensor exhibits excellent mechanical reliability, maintaining stable output signals over 10^6^ loading cycles without noticeable degradation, as illustrated in Figure 3g, highlighting its superior fatigue resistance. This performance substantially surpasses the cycling stability of transferred PZT devices (∼5000 cycles) [6] and compositionally graded PZT (∼12 000 cycles) [45], indicating markedly enhanced mechanical durability and operational reliability. Moreover, both the output voltage and power exhibit strong dependence on external load resistance, with an optimal impedance matching condition identified for maximizing power output (Figure 3h). The output power of 8.1 µW is substantially higher than previously reported values of 3.6 [25] and 5.3 µW [46], highlighting its superior energy harvesting performance. Such output characteristics are important performance considerations for piezoelectric mechano‐electrical conversion, and quantitative metrics for systematically evaluating piezoelectric energy conversion performance have been established in previous studies [47, 48]. These characteristics, together with the intrinsic flexibility and high sensitivity of the freestanding film, make it highly promising for wearable sensing applications.

### 2.3 Wearable Physiological Motion and Acoustic Detection

To demonstrate its practical applicability in wearable and human‐machine interaction scenarios, the device was further evaluated under various physiological motions and acoustic stimuli. As demonstrated in Figure 4a–e, distinct and stable voltage signals are generated in response to arm bending, finger pressing, hand tapping, foot stepping, and wrist bending, confirming reliable mechanical‐to‐electrical signal transduction across multiple body sites. In addition, the device also exhibits clear and distinguishable responses during hand grasping and releasing cycles in Figure 4f, demonstrating its capability to resolve dynamic motion states with high fidelity. As a representative wearable application, continuous hand tapping was performed to further evaluate the operational stability of the device over 2000 s (Figure S7). The device maintained a stable output throughout the measurement, with the positive voltage consistently reaching approximately 6 V. Representative signals recorded at the early (37–39 s), middle (871–873 s), and late (1930–1932 s) stages exhibit comparable output amplitudes and reproducible waveforms. No obvious baseline shift or progressive signal attenuation is observed, further supporting the operational stability of the device under prolonged repetitive mechanical stimulation. Importantly, beyond biomechanical sensing, the device also enables acoustic signal detection, as demonstrated by the well‐defined voltage waveform corresponding to the spoken words “Hello” in Figure 4g. The observed signal characteristics highlight the system's potential for voice recognition and acoustic monitoring, extending its functionality from mechanical sensing to multimodal human‐machine interaction.

**FIGURE 4:**
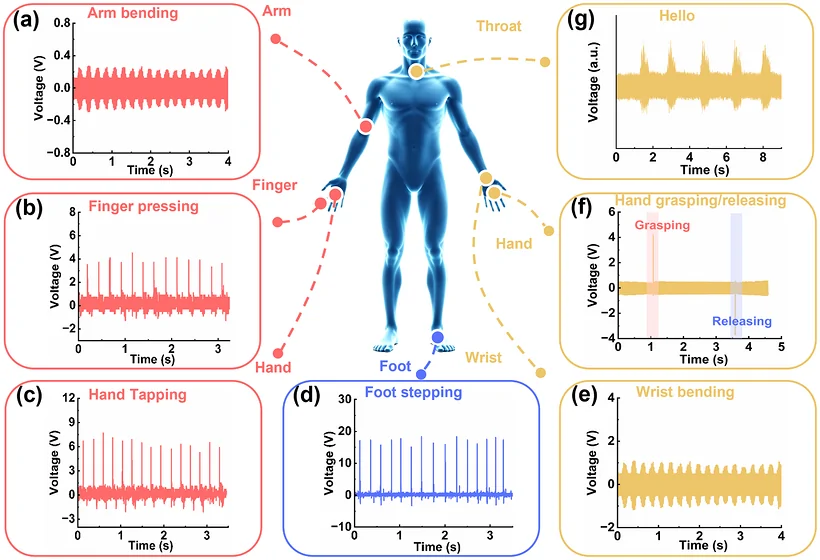
(a–e) Output voltage under diverse human motions, including arm bending, finger pressing, hand tapping, foot stepping, and wrist bending, demonstrating stable and repeatable mechanical‐to‐electrical transduction. (f) Output signals during hand grasping and releasing, exhibiting clear voltage responses. (g) Acoustic response of the device during speech “Hello”, exhibiting well‐defined waveform features for voice detection and signal analysis.

Beyond mechanical sensing, the PZT sensor exhibits excellent capability for acoustic signal transduction, enabling advanced sound recognition functionality. Figure 5a demonstrates that the sensor yields distinct and stable frequency‐dependent voltage outputs in response to acoustic excitations across the 400–3000 Hz range. To validate this capability under realistic conditions, we extended the analysis to acoustic inputs, using the piano note “fa” as a representative case for time‐ and frequency‐domain characterization. As displayed in Figure 5b, the time‐domain response exhibits a well‐defined waveform with high reproducibility, while the corresponding fast Fourier transform (FFT) spectrum in Figure 5c clearly resolves the fundamental frequency (*f*_0_) along with multiple harmonic components [49]. The additional peaks at 50 Hz and its harmonics are attributed to power‐line interference, confirmed by FFT analysis of the background signal in Figure S8, highlighting the sensor's high sensitivity and spectral resolution. Figures S9–S12 present stable voltage responses to various musical notes generated from different musical instruments. Furthermore, the short‐time Fourier transform (STFT) spectrogram (Figure 5d) reveals the temporal evolution of frequency components [50, 51]. Notably, the stable distribution and temporal persistence of the fundamental frequency and its harmonics reflect the high fidelity and reliability of the acoustic signal transduction. To further evaluate its capability for musical signal recognition, the sensor was tested with piano tones spanning the diatonic scale, as presented in Figure 5e.

**FIGURE 5:**
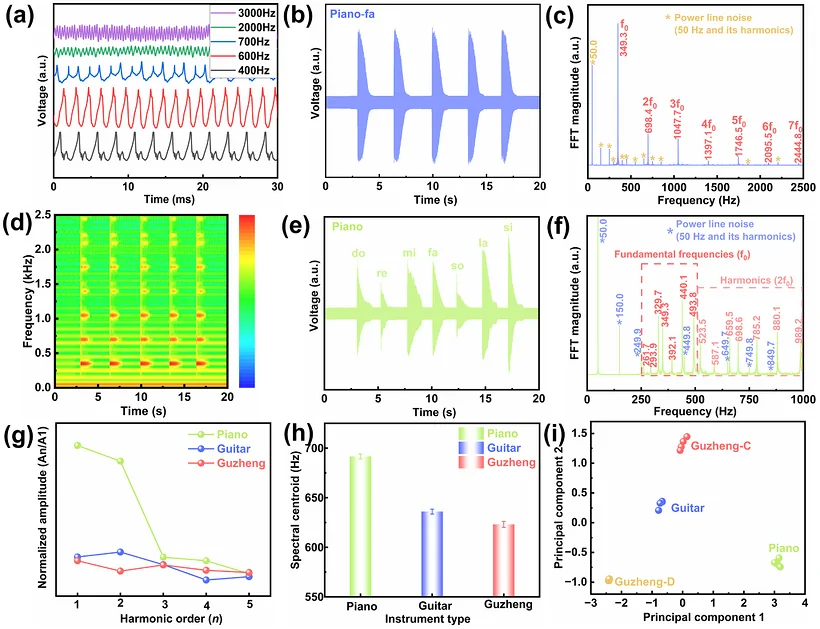
(a) Output voltage responses of the device under acoustic excitation across different frequencies (400–3000 Hz). (b) Time‐domain voltage response of the device to a representative piano note “fa”. (c) Corresponding FFT spectrum of the acoustic response, resolving the fundamental frequency and higher‐order harmonics of the input signal, together with observable background power‐line interference components. (d) STFT spectrogram illustrating the time‐frequency evolution of the piano signal. (e) Output voltage responses corresponding to a sequence of piano notes spanning the diatonic scale (do–si). (f) FFT spectra of the corresponding notes, revealing clearly separated fundamental frequencies (*f*_0_) and characteristic harmonic distributions. (g) Normalized harmonic amplitude distributions versus harmonic order for different instruments (piano, guitar, and guzheng). (h) Statistical comparison of spectral centroid distributions across different instruments. (i) PCA of the extracted acoustic features, revealing well‐separated clusters corresponding to different instruments and tonal conditions.

Distinct voltage response patterns are observed for individual musical notes, while the corresponding FFT spectra in Figure 5f clearly reveal the fundamental frequencies along with well‐preserved harmonic structures, demonstrating accurate and reliable frequency discrimination. Importantly, the sensor enables differentiation of musical instruments through harmonic feature analysis. As displayed in Figure 5g, the normalized amplitudes of higher‐order harmonics exhibit distinct distributions among different instruments (piano, guitar, and guzheng), reflecting their intrinsic timbre characteristics. This distinction is further quantified using spectral centroid analysis in Figure 5h, where each instrument exhibits distinct values, reflecting its unique spectral energy distribution and enabling reliable instrument classification. To facilitate high‐level pattern recognition, PCA was conducted on the extracted acoustic features [52, 53]. As demonstrated in Figure 5i, clear clusters corresponding to different instruments and tonal conditions are well separated in the reduced‐dimensional feature space, indicating robust and reliable classification capability of the system. Collectively, these results demonstrate that the freestanding PZT‐based sensor serves not only as a high‐performance acoustic sensing platform, but also as an integrated system for signal analysis and intelligent recognition, highlighting its potential for self‐powered auditory systems and human‐machine interfacing technologies.

## 3 Conclusions

In summary, a freestanding PZT‐based film has been developed to overcome substrate‐induced clamping effects. The release of substrate constraints leads to significantly enhanced properties, including a 1.4‐fold increase in *P*_r_ (54 vs. 40 µC cm^−^^2^), a 2.2‐fold enhancement in *d*_33_ (390 vs. 174 pm V^−^^1^), and a 1.5‐fold improvement in *ε*_r_ (1207 vs. 805). Benefiting from these superior properties, the device delivers ultrafast voltage pulses as short as 500 µs and excellent electrical outputs under compressive and bending deformations. Furthermore, the sensor exhibits excellent operational stability, high sensitivity, and outstanding mechanical adaptability, highlighting its potential for diverse wearable sensing applications. Importantly, the self‐powered system enables high‐fidelity acoustic sensing, while spectral feature extraction combined with principal component analysis enables reliable discrimination of acoustic signatures from different instruments. We note that the present PZT system contains lead, which may raise concerns for skin‐contact wearable applications. Although the device is encapsulated with PI tape as a physical barrier, long‐term safety under sweat exposure and mechanical abrasion requires further evaluation. Nevertheless, the core innovation—a freestanding architecture enabled by van der Waals‐assisted delamination—does not rely on lead‐based chemistry. The demonstrated structural paradigm is seamlessly transferable to emerging lead‐free piezoelectric systems, such as potassium sodium niobate and bismuth sodium titanate thin films, where comparable strain‐release benefits are expected. This work thus provides a general strategy for enhancing the electromechanical performance of flexible ferroelectrics and establishes a multifunctional self‐powered platform for integrated sensing, energy harvesting, and acoustic recognition, paving the way for next‐generation wearable electronics and smart acoustic technologies.

## 4 Experimental Section

### 4.1 Precursor Preparation

An LNO precursor solution was prepared by dissolving stoichiometric amounts of lanthanum nitrate (La(NO_3_)_3_·6H_2_O, Aladdin, 99.99%) and nickel nitrate (Ni(NO_3_)_2_·6H_2_O, Aladdin, 99%) in a mixed solvent of 2‐methoxyethanol (C_3_H_8_O_2_, Sinopharm Chemical Reagent, 99.5%) and acetic acid (C_2_H_4_O_2_, Sinopharm Chemical Reagent, 99.8%). The solution was stirred for 12 h to obtain a homogeneous precursor solution. A 0.4 mol L^−1^ PZT precursor sol was prepared via a sol–gel method. To suppress the formation of pyrochlore and fluorite secondary phases, a 10 mol% excess of Pb was employed. Initially, tetrabutyl titanate (C_16_H_36_O_4_Ti, Aladdin, 98%) was chelated with acetylacetone (C_5_H_8_O_2_, Sinopharm Chemical Reagent, 99%) under continuous stirring at room temperature for 4 h to form a stable titanium‐containing solution (Solution A). Subsequently, lead(II) acetate trihydrate (C_4_H_6_O_4_Pb·3H_2_O, Sinopharm Chemical Reagent, 99%) was dissolved in C_3_H_8_O_2_ at 60°C, followed by the addition of C_2_H_4_O_2_ to facilitate complete dissolution. After cooling to room temperature, the resulting solution was used as the lead precursor solution (Solution B). Separately, zirconium n‐propoxide (C_12_H_28_O_4_Zr, Aladdin, 70%) was dissolved in C_3_H_8_O_2_ under stirring at room temperature to obtain Solution C. Finally, Solutions B and C were sequentially added to Solution A under continuous stirring, followed by stirring for an additional 12 h to obtain the final PZT precursor solution.

### 4.2 Film Preparation

Flexible mica substrates were sequentially cleaned with acetone, ethanol, and deionized water, followed by drying. A Pt bottom electrode was deposited onto the mica substrates by DC magnetron sputtering (Q150T S, Quorum) in Ar, and the Pt‐coated substrates were subsequently pre‐annealed at 600°C. The 0.1 mol L^−1^ LNO precursor solution was spin‐coated onto the Pt/mica substrates at 3000 rpm for 30 s. The deposited films were dried at 350°C for 5 min and subsequently annealed at 700°C for 15 min to achieve crystallization. This spin‐coating–pyrolysis–annealing cycle was repeated four times. Then, the PZT precursor solution was spin‐coated onto the pre‐treated substrates at 3000 rpm for 30 s, followed by pyrolysis at 250°C for 20 min and rapid thermal annealing at 650°C for 10 min to crystallize the film. This procedure was repeated 6, 8, and 10 times to prepare 6‐, 8‐, and 10‐layer PZT films, respectively. For electrical characterization, Pt top electrodes with a diameter of 200 µm were deposited onto the as‐prepared PZT films by DC sputtering.

Fabrication of the PZT‐based sensor: Pt interdigital top electrodes were fabricated on the PZT film via magnetron sputtering through a patterned mask. The devices were encapsulated with PI tape to ensure mechanical integrity and environmental stability while preserving flexibility, and more importantly, to act as an effective barrier against potential Pb leakage. In addition, electrical poling was performed at room temperature under a constant bias voltage of 25 V for 24 h to align ferroelectric domains and increase the piezoelectric response.

### 4.3 Characterization and Instrumentation

The crystal structure of the films was analysed by X‐ray diffraction (XRD, Bruker D8 Advance) using Cu Kα radiation. Surface morphology, cross‐sectional microstructure, and elemental composition were studied by field‐emission scanning electron microscopy (FE‐SEM, ZEISS GeminiSEM360) equipped with energy‐dispersive X‐ray spectroscopy (EDS). The average grain size was statistically determined using Nano Measurement 1.2.0 software. Ferroelectric properties were characterized using a ferroelectric tester (Radiant Precision Multiferroic). Local piezoelectric responses, ferroelectric switching behaviors, and domain structures were characterized using piezoresponse force microscopy (PFM; MFP‐3D Origin+, Oxford, USA). Electrical output signals were measured via an oscilloscope (MDO34‐3‐BW‐200, Tektronix). Finite‐element simulations were conducted using COMSOL Multiphysics.

### 4.4 Acoustic Signal Processing and PCA Analysis

Time‐ and frequency‐domain analyses were performed on the measured voltage signals to derive acoustic features. FFT and STFT were adopted to resolve frequency components and their temporal evolution, respectively. Additionally, key descriptors, including harmonic ratios and spectral centroid, were extracted to quantitatively characterize timbral characteristics. PCA was then performed on the normalized features for dimensionality reduction and cluster identification. The leading principal components captured the majority of variance, clearly separating different instruments and tonal conditions in the reduced space.

## Author Contributions

H.P.: conceptualization, methodology, investigation, writing – original draft, data curation, formal analysis; **Z. X**., **R.L**., and **J.P**.: data curation, visualization, validation, software; **C.Y**., **S.H**., and **M.W**.: supervision, funding acquisition, project administration, writing – review & editing, resources.

## Funding

Taishan Scholar Foundation of Shandong Province (Grant No. tstp20221130); Shandong Province Key Fundamental Research Program (Grant No. ZR2026ZD42); University of Jinan Major Science and Technology Projects and Landmark Achievements Cultivation Program (XKY2508).

## Conflicts of Interest

The authors declare no conflicts of interest.

## Supporting information




**Supporting File**: advs78091‐sup‐0001‐SuppMat.docx.

## Data Availability

The data that support the findings of this study are available on request from the corresponding author. The data are not publicly available due to privacy or ethical restrictions.
