# Supplementary material for: Freestanding PZT Films for Integrated Wearable Sensing and Intelligent Acoustic Recognition
Source: Adv Sci (Weinh). 2026 Oct 14:e78091. Online ahead of print. doi: 10.1002/advs.78091 (PMC13668060; doi:10.1002/advs.78091)
Supplement: Supplementary file 1 [file ADVS-9999-e78091-s001.docx]

Supporting Information

**Freestanding PZT Films for Integrated Wearable Sensing and Intelligent Acoustic Recognition**

*Hailong Pan*, *Zhichao Xu*, *Rongxiao Liu*, *Jianmei Pei*, *Changhong Yang**, *Shifeng Huang*, *Michael Wübbenhorst*

**Figure S1** EDS elemental mapping of the Pt/LNO/PZT heterostructure.

**Figure S2** DC bias-dependent *d*_33_ and phase loops for the clamped film.

**Figure S3** (a, b) *P*–*E* hysteresis loops under different applied voltages and frequencies. (c) Polarization evolution as a function of cycling. (d) Comparison of *P*–*E* loops before and after 10^9^ cycles.

**Figure S4** *P–E* loops of Pt/LNO/PZT heterostructures with 6-, 8-, and 10-layer PZT films in the (a) clamped and (b) freestanding states. (c) Comparison of *P*_r_, *P*_s_, and *E*_c_ for the clamped and freestanding heterostructures with different PZT thicknesses. Electric field dependent *ε*_r_ of the corresponding heterostructures under the (d) clamped and (e) freestanding conditions. (f) Comparison of *ε*_r_ for different PZT film thicknesses under clamped and freestanding conditions.

**Figure S5** Output voltage of the clamped film under bending deformation at 60°.

**Figure S6** FEA of strain distribution in the device under different bending angles.

**Figure S7** Operational stability of the device under continuous hand tapping for 2000 s.

**Figure S8** FFT spectrum of the background noise.

**Figure S9** Output voltage signals of the sensor under excitation by piano notes (do–si scale).

**Figure S10** Output voltage signals of the sensor under excitation by guitar notes (do–si scale).

**Figure S11** Output voltage signals of the sensor under excitation by guzheng notes in the C major scale (do–si).

**Figure S12** Output voltage signals of the sensor under excitation by guzheng notes in the D major scale (do–si).

**Table S1** Comparison of *d*_33_ and *P*_r_ in PZT thin films. SRO: SrRuO_3_; STO: SrTiO_3_; LNO: LaNiO_3_; LSCO: La_0.5_Sr_0.5_CoO_3_; ZRC: Zirconia Ribbon Ceramics; LSMO: La_0.7_Sr_0.3_MnO_3_; PLD: Pulsed Laser Deposition; CSD: Chemical solution deposition.

| Thin-film structure | Preparation method | Thickness (nm) | *d*_33_  (pm V^–1^) | *P*_r_  (μC cm^–2^) | References |
| --- | --- | --- | --- | --- | --- |
| PZT/SRO/STO | PLD | 400 | 150.6 | 25 | ^[1]^ |
| PZT/LNO/Si | CSD | 900 | -- | 18 | ^[2]^ |
| PZT/STO | PLD | 250 | 83 | 47 | ^[3]^ |
| PZT/SRO/STO | PLD | 160 | 157 | -- | ^[4]^ |
| PZT/LSCO/STO/Si | CSD | 120 | 200 | 41 | ^[5]^ |
| PZT/Pt/Ti/SiO_2_/Si | CSD | 1200 | 108 | 14 | ^[6]^ |
| PZT/Pt/TiO_2_/Ti/SiO_2_/Si | CSD | 1000 | 250 | -- | ^[7]^ |
| PZT/Pt/Ti/SiO_2_/Si | CSD | 1600 | 98 | 15 | ^[8]^ |
| PZT/Pt/TiO_2_/SiO_2_/Si | CSD | 1000 | 170 | 30 | ^[9]^ |
| PZT/LNO/ZRC | CSD | 2100 | 271 | 40 | ^[10]^ |
| PZT/LNO/Mica | CSD | 1800 | 210 | 49 | ^[11]^ |
| PZT/LSMO/Mica | PLD | 80 | 220 | 14 | ^[12]^ |
| PZT/Mica | CSD | 2000 | 130 | -- | ^[13]^ |
| Polycrystalline PZT | CSD | 700 | 474 | 43 | ^[14]^ |
| Single crystalline PZT (001) | PLD | 250 | 217 | 27 | ^[15]^ |
| Single crystalline PZT (111) | PLD | 250 | 585 | 36 | ^[15]^ |
| PZT/LNO/Pt/Mica | CSD | 600 | 174 | 40 | This work |
| PZT/LNO/Pt | CSD | 600 | 390 | 54 | This work |

**References**

1. M. Acharya, D. Lou, A. Fernandez, J. Kim, Z. Tian, and L. W. Martin, “Direct Measurement of Inverse Piezoelectric Effects in Thin Films Using Laser Doppler Vibrometry,” *Physical Review Applied* *20*, no. 1 (2023): 014017, https://doi.org/10.1103/PhysRevApplied.20.014017.

2. J. Zhang, W. Jia, Q. Zhang, et al., “Controlled Spalling and Flexible Integration of PZT Film Based on LaNiO_3_ Buffer Layer,” *Ceramics International* *45*, no. 5 (2019): 6373–6379, https://doi.org/10.1016/j.ceramint.2018.12.123.

3. M. D. Nguyen, M. Dekkers, E. Houwman, et al., “Misfit Strain Dependence of Ferroelectric and Piezoelectric Properties of Clamped (001) Epitaxial Pb(Zr_0.52_,Ti_0.48_)O_3_ Thin Films,” *Applied Physics Letters* *99*, no. 25 (2011): 252904, https://doi.org/10.1063/1.3669527.

4. V. Nagarajan, C. L. Jia, H. Kohlstedt, et al., “Misfit Dislocations in Nanoscale Ferroelectric Heterostructures,” *Applied Physics Letters* *86*, no. 19 (2005): 192910, https://doi.org/10.1063/1.1922579.

5. Y. Wang, C. Ganpule, B. T. Liu, et al., “Epitaxial Ferroelectric Pb(Zr, Ti)O_3_ Thin Films on Si Using SrTiO_3_ Template Layers,” *Applied Physics Letters* *80*, no. 1 (2002): 97–99, https://doi.org/10.1063/1.1428413.

6. T. Iijima, S. Ito, and H. Matsuda, “Evaluation of Longitudinal Displacement for Lead Zirconate Titanate Films,” *Japanese Journal of Applied Physics* *41*, no. 11S (2002): 6735, https://doi.org/10.1143/JJAP.41.6735.

7. D. Balma, A. Mazzalai, N. Chidambaram, et al., “High Piezoelectric Longitudinal Coefficients in Sol–Gel PZT Thin Film Multilayers,” *Journal of the American Ceramic Society* *97*, no. 7 (2014): 2069–2075, https://doi.org/10.1111/jace.12894.

8. L. Lian, and N. R. Sottos, “Effects of Thickness on the Piezoelectric and Dielectric Properties of Lead Zirconate Titanate Thin Films,” *Journal of Applied Physics* *87*, no. 8 (2000): 3941–3949, https://doi.org/10.1063/1.372439.

9. V. Kovacova, N. Vaxelaire, G. Le Rhun, P. Gergaud, T. Schmitz-Kempen, and E. Defay, “Correlation between Electric-Field-Induced Phase Transition and Piezoelectricity in Lead Zirconate Titanate Films,” *Physical Review B* *90*, no. 14 (2014): 140101, https://doi.org/10.1103/PhysRevB.90.140101.

10. Y. Liu, L. Ding, L. Dai, et al., “All-Ceramic Flexible Piezoelectric Energy Harvester,” *Advanced Functional Materials* *32*, no. 52 (2022): 2209297, https://doi.org/10.1002/adfm.202209297.

11. D. Wang, G. Yuan, G. Hao, and Y. Wang, “All-Inorganic Flexible Piezoelectric Energy Harvester Enabled by Two-Dimensional Mica,” *Nano Energy* *43* (2018): 351–358, https://doi.org/10.1016/j.nanoen.2017.11.037.

12. R. Zhang, Y. Ding, N. Liu, et al., “High Macroscopic Piezoelectric *d*_33_ of the Nm-Thick Flexible PZT Ferroelectric Film,” *Journal of Materials Science: Materials in Electronics* *35*, no. 4 (2024): 298, https://doi.org/10.1007/s10854-024-12040-6.

13. S. Liu, D. Zou, X. Yu, Z. Wang, and Z. Yang, “Transfer-Free PZT Thin Films for Flexible Nanogenerators Derived from a Single-Step Modified Sol–Gel Process on 2D Mica,” *ACS Applied Materials & Interfaces* *12*, no. 49 (2020): 54991–54999, https://doi.org/10.1021/acsami.0c16973.

14. Q. Xu, M. Jia, P. Zhou, et al., “High‐Performance Ultrasensitive Flexible Piezoelectric Thin Film Sensors via a Cost‐Effective Transfer Strategy,” *Advanced Functional Materials* *35*, no. 4 (2025): 2414211, https://doi.org/10.1002/adfm.202414211.

15. Z. Ren, S. Deng, J. Shao, et al., “Ultrahigh-Power-Density Flexible Piezoelectric Energy Harvester Based on Freestanding Ferroelectric Oxide Thin Films,” *Nature Communications* *16*, no. 1 (2025): 3192, https://doi.org/10.1038/s41467-025-58386-1.
